# Serial magnetic resonance imaging of splenomegaly in the *Trypanosoma brucei* infected mouse

**DOI:** 10.1371/journal.pntd.0010962

**Published:** 2022-12-07

**Authors:** Samantha Paterson, William Matthew Holmes, Jean Rodgers

**Affiliations:** 1 Institute of Medical and Biological Engineering, School of Mechanical Engineering, University of Leeds, Leeds, United Kingdom; 2 Glasgow Experimental MRI Centre, Institute of Neuroscience and Psychology, University of Glasgow, Glasgow, United Kingdom; 3 Institute of Biodiversity, Animal Health & Comparative Medicine, College of Medical, Veterinary & Life Sciences, University of Glasgow, Glasgow, United Kingdom; Hunter College, CUNY, UNITED STATES

## Abstract

Splenomegaly, an enlargement of the spleen, is a known clinical sign of the parasitic disease, human African trypanosomiasis. This study follows the development of splenomegaly in a group of mice over multiple infection points, using a non-invasive imaging modality, magnetic resonance imaging (MRI). CD-1 mice infected with GVR35 *T*.*b*. *brucei* demonstrated a significant increase in spleen size from day 7 post-infection, with changes in the spleen tracked in individual animals over five time points. At the final time point, the mean spleen weight calculated using the spleen volume from the MR images was compared with the post-mortem gross spleen weight. No significant difference was detected between the two methods (1.62 ± 0.06g using MRI and 1.51 ± 0.04g gross weight, p = 0.554). Haematology and histological analysis were also performed, giving additional insight into splenomegaly for the GVR35 strain of infection. The study demonstrates that MRI is a useful tool when examining changes in organ volume throughout HAT infection and may be applicable in the investigation of a range of conditions where changes in organ volume occur and MRI has not been used previously.

## Author summary

Although splenomegaly (the enlargement of the spleen) has been explored previously in mice infected with human African trypanosomiasis (HAT), a parasitic disease originating in Africa, it has never been examined in individual mice over time. This study is the first to do this using magnetic resonance imaging (MRI). By scanning mice infected with trypanosomes, we were able to examine how the spleen size and shape changed over the course of the infection. We were also able to show the enlargement in spleen size before the clinical signs became apparent, demonstrating that MRI could be useful for examining changes in the organs in the body following the onset of disease.

## Introduction

Human African trypanosomiasis (HAT) is a parasitic disease found in Sub-Saharan Africa. Trypanosomes are transmitted to the host via the bite of a tsetse fly vector. The species *Trypanosoma brucei* can be split into three sub-species. Two of these can infect humans, *Trypanosoma brucei (T*.*b*.*) rhodesiense*, found in East and Southern Africa, and *T*.*b*. *gambiense*, which is mainly found in West Africa [1]. The third subspecies, *T*.*b*. *brucei*, can infect cattle and other animals. In *T*.*b*. *rhodesiense*, the parasite is also found in antelope and cattle reservoirs. By contrast, for *T*.*b*. *gambiense*, the parasite reservoir is almost exclusively within humans. The disease progresses over two stages, the haemolymphatic or early-stage, and the encephalitic or late-stage. In the early stage of the disease, the parasites reside initially in the blood and lymph nodes, and then disseminate into many organs including the skin, spleen, liver, heart, and eyes [2]. The disease is classified as late-stage when the parasite can be detected in the central nervous system (CNS) or there is a raised white blood cell count in the cerebral spinal fluid (CSF) [3]. There are many non-specific clinical signs associated with trypanosome infection such as headaches, fatigue, fever, lymphadenopathy, sleep disturbances, cardiac dysfunction, hepatomegaly and splenomegaly.

The development of splenomegaly, or enlargement of the spleen, is a recognised clinical sign resulting from trypanosome infection in humans [4–6] and rodents [7–9]. The spleen is the largest secondary immune organ in the body and is responsible for initiating the immune response to blood-borne antigens [10]. Splenomegaly can occur due to a wide range of diseases or infections including AIDS, malaria, sickle cell disease and lymphoma amongst others. The resulting increase in the size of the spleen is highly dependent on the mechanism involved. These mechanisms include congestion, infiltration and increased splenic function [11].

To date there has been no exploration of splenomegaly development in HAT using non-invasive measurements, meaning it has not been tracked in a single animal. One method is the use of magnetic resonance imaging (MRI). MRI uses a combination of a strong magnetic field and radio waves to produce anatomical images of the body. The protons in the body align with the magnetic field and after a radiofrequency pulse is applied, the direction of the protons flip. The time it takes these protons to return to align with the magnetic field (known as relaxation) determines the contrast that is seen in the images. Two types of relaxation are called T_1_ and T_2_ relaxation and the images produced from these effects differ depending on the properties of the tissue involved.

This study aims to explore the development of splenomegaly in a well characterised murine model of HAT [12] for the first time at multiple infection time points in individual animals using serial MR scanning, histology, and haematology investigation.

## Materials & methods

### Ethics statement

All experiments were approved by the local University of Glasgow ethics review board and the UK Home Office Animals (Scientific Procedures) Act 1986. All methods were carried out in **accordance with relevant guidelines and regulations**. The study was carried out in compliance with the ARRIVE guidelines.

### Animals and infections

The well-established *T*. *b*. *brucei* GVR35 mouse model of human African trypanosomiasis was used in this study. Experiments were performed on female CD-1 mice (n = 6 infected, n = 5 control), with a body weight of 30–38g. All animals were sourced from the Charles River Lab. A passage mouse was infected with 2 x 10^4^ *T*. *b*. *brucei* GVR 35 C1.9 parasites, taken from frozen stabilate, in 100μL phosphate buffered saline glucose (PBSG-G) by intraperitoneal injection. At the first parasitaemic peak, on day 7-post infection, the passage animal was culled and exsanguinated. The blood was then used to infect mice in the experimental group (n = 6) as described previously [12].

### Magnetic resonance imaging

**Fig 1:**
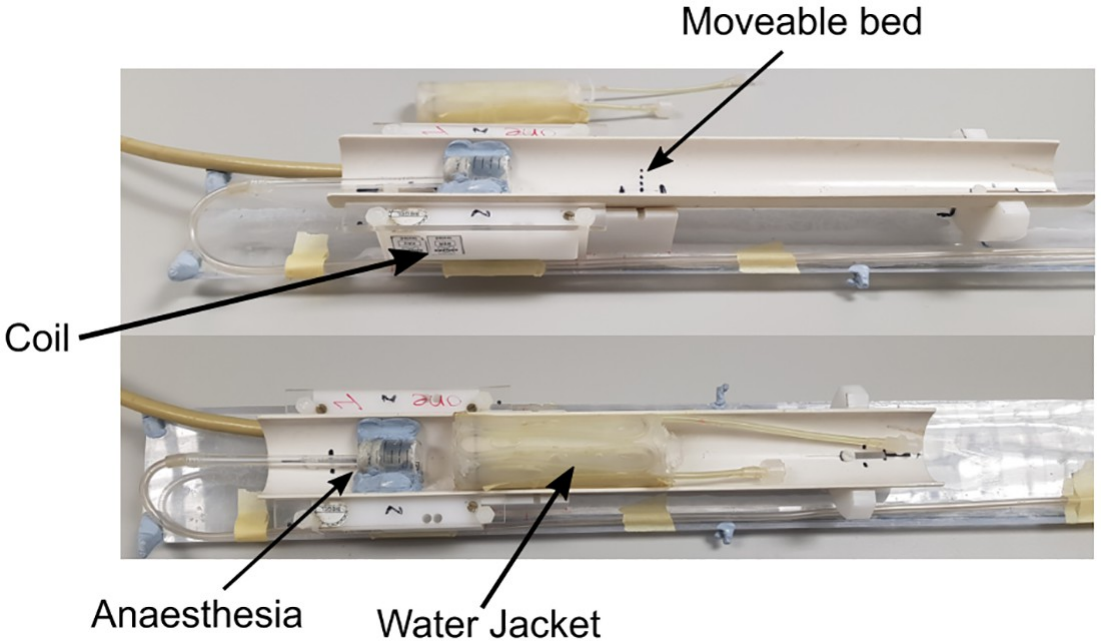
Scanning setup. A custom-made cradle was designed for full body mouse imaging using a rat head surface coil. A water jacket is placed on top of the mouse to maintain body temperature in the scanner. Anaesthetic is fed through a tube to the mouse during the scan session. A moveable half cylindrical bed is placed on top of the 4-channel rat head surface coil to allow optimal positioning of the mouse in the scanner. This also protects the coil.

Experiments were performed on a horizontal 7 T Bruker Pharmascan Avance III system (300 MHz). A Bruker BGA9 imaging gradient insert (300 mT m^-1^) was used to provide linear magnetic field gradient pulses. A 72mm birdcage radiofrequency (RF) volume resonator was used to transmit and a 4-channel rat brain phase array coil to receive. The animals were anaesthetized in a chamber using 5% isoflurane and a 30: 70 O_2_:N_2_O ratio before being transferred to the MRI scanner and maintained on 2–3% isoflurane. To facilitate acquisition of high-quality images of the whole mouse abdomen, a custom-made cradle was used, containing an inverted 4-channel rat brain surface coil (Fig 1).

T_1_ and T_2_ scans covering the abdomen were run for each animal using prospective respiratory gating. For T_1_ imaging, a FLASH (fast low angle shot) sequence was used with the following parameters: TE = 2.534ms, TR = 600ms, NA = 4, RF pulse duration of 0.9ms, flip angle = 30 degrees. A RARE (rapid acquisition with refocused echoes) sequence was used for the T_2_ weighted images with the following parameters: TE = 13.75ms, Effective TE = 55ms, RARE Factor = 8, TR = 8000ms, NA = 1. For both T_1_ and T_2_: FOV = 5x5cm. Matrix size = 200x200 with an in-plane resolution of 0.025x0.025cm. Thirty slices of 1mm thickness were used which covered the whole body of the mouse. Scan time was approximately 4 minutes for T_2_ and approximately 8 minutes for T_1_. Total scan time was less than 20 minutes including an initial localiser scan.

Animals were serially scanned at days 0, 7, 14, 21 and 28 post-infection (pi). At day 28, the mice were culled, and spleens excised, weighed, and processed for histology. The procedures were repeated using an uninfected control group. At each time point the mice were weighed, parasitaemia levels checked and recorded (if applicable), with blood taken for haematological analysis.

### Haematology

Blood samples (100μL) were taken from all animals by tail bleeding at each time point and exsanguination at the experimental endpoint. These samples were processed by University of Glasgow Veterinary Diagnostic Services. The following parameters were assessed: red blood cells (RBC), haemoglobin (Hb), haematocrit (HCT %), white blood cells (WBC), neutrophils, lymphocytes, and monocytes. Only samples from day 0 and the endpoint were successfully processed due to clotting issues in the samples in between these points.

### Histopathology

At the end point of the experiment, spleens were taken and fixed in 4% neutral buffered formalin. The tissue was then paraffin wax embedded and 3 μm sections prepared for histological analysis. Haematoxylin and Eosin (H & E) stained sections were used to assess the histopathology, while immunohistochemistry was performed to detect T-cells (rabbit anti-CD3 polyclonal; 1:100 dilution; DAKO) and B-cells (rabbit anti-PAX5 monoclonal; 1:500 dilution; Abcam) following a standard peroxidase anti-peroxidase protocol using the Dako EnVision system and DAB visualisation. All processing, sectioning, and staining was performed by University of Glasgow Veterinary Diagnostic Services.

### Data processing and statistical analysis

All data were analysed using in-house MATLAB code. All image slices were examined and if the slice contained the spleen, a region of interest was drawn around the tissue. The overall volume of the spleen was calculated as the sum of the volumes of the regions of interest from each slice.

The mass of the spleen was calculated using the following equation:

m=ρV (1)

where ρ is density (g/mm^3^), m is mass (g) and V is MRI volume (mm^3^). The density of the spleen was taken to be 0.001089 g/mm^3^ [13] and the volume from the day 28 pi datasets were used for calculating the mass.

Statistical analyses were performed using Minitab 17 (Minitab inc.). Analysis of variance methods were used to explore the data from each infection time point. To detect differences between the groups a general linear model was employed followed by Tukey’s multiple range test. Differences between groups were considered significant when p-values were less than 0.05.

## Results

### Magnetic resonance imaging

**Fig 2:**
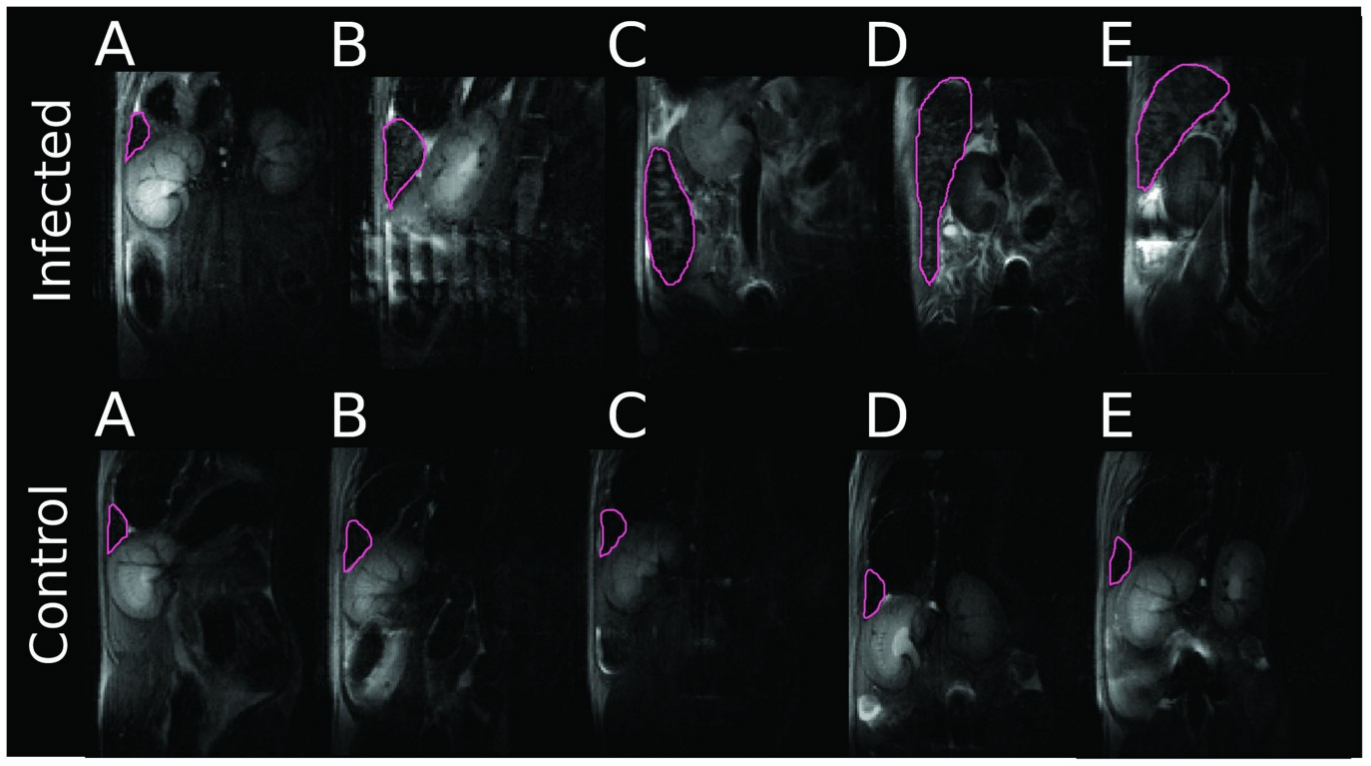
MR images of two mice. T_2_ weighted images of one infected and one uninfected (control) mouse throughout the experiment. Animals were scanned at days 0 (A), 7 (B), 14 (C), 21 (D) and 28 (E) post-infection or similar timepoints in control (uninfected). Increased spleen size can be seen in the infected animal at all timepoints post-infection (p<0.005). No splenomegaly was apparent in the control mouse. The spleen is outlined in pink.

Fig 2 shows an example of serial T_2_ MR images from one mouse acquired at approximately the same spatial position. The infected mouse group showed a clear increase in spleen volume from day 7 pi onward, with the size, shape and position of the spleen appearing different for each animal, but all displaying a clear enlargement. On physical examination, the enlarged spleen was observed in mice from day 14 pi. By comparison, for the control animals, the typical triangular cross-section of the spleen was observed at all time points, with no change in size detected. A full set of MR images from one scan of a healthy mouse and one infected mouse are shown in S1 and S2 Figs.

**Fig 3:**
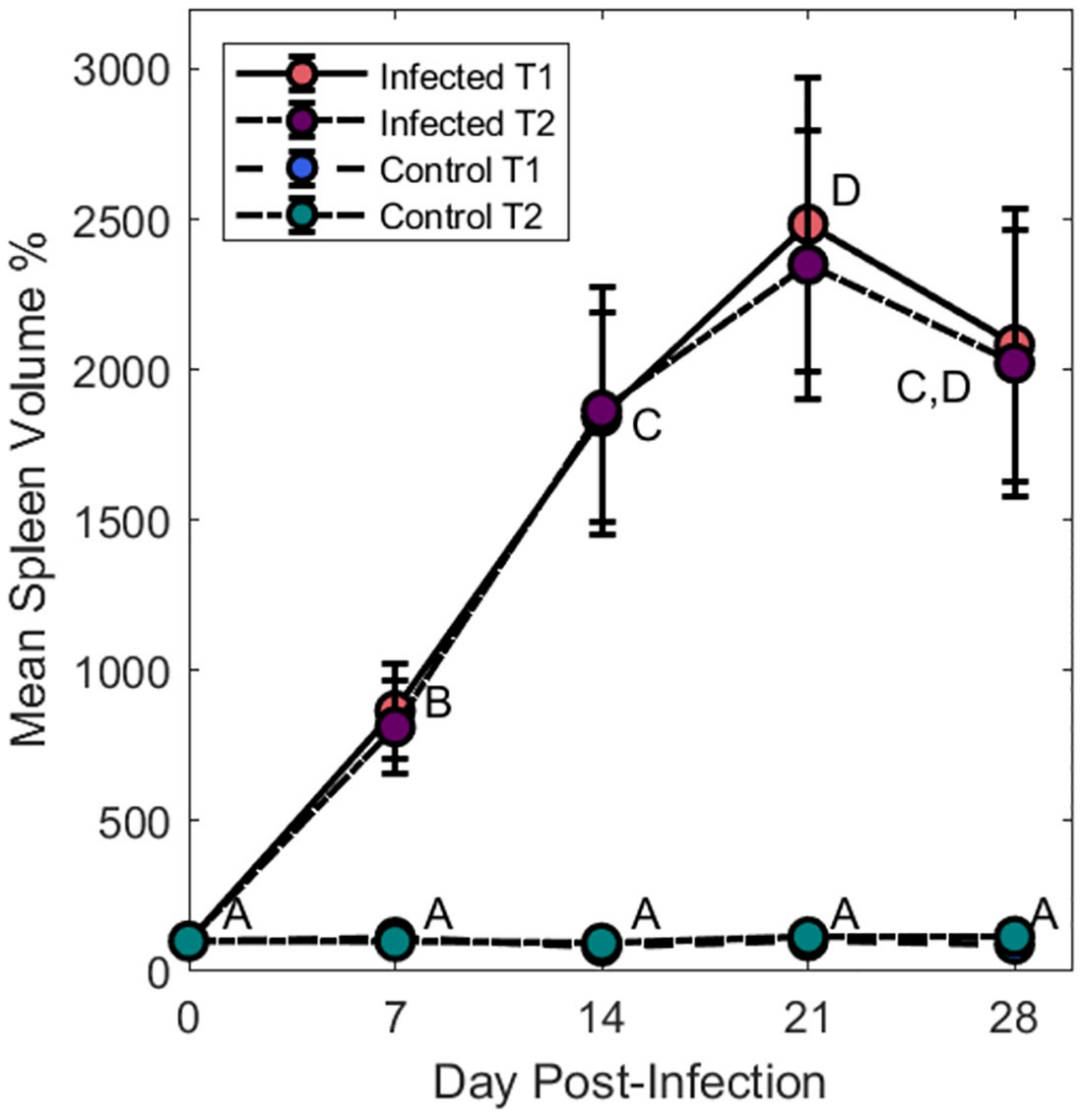
Comparison of spleen volume. Comparison of the mean spleen volumes demonstrates a large change in volume for the infected animals, with both T_1_ and T_2_ data shown. The mean spleen volume for the control groups using T_1_ and T_2_ are not significantly different (p > 0.05) and are overlayed. Spleen volume is shown as a percentage of the volume measured at day 0. Splenomegaly was apparent after day 0 pi with the peak increase in volume at day 21pi for the T_1_ weighted data at 2483% of the original volume and 2349% for the T_2_ weighted data. A significant (p < 0.05) stepwise increase can be seen following infection until day 21pi. All infected spleen volumes are significantly higher (p<0.05) compared with their pre-infection states. The control group shows no significant change (p > 0.05, ~1% change in volume) over the time course of the experiment. The means of groups that do not share a letter are significantly different from each other (p < 0.05).

There was no statistical significance between the spleen volumes measured using the T_1_ or T_2_ weighted sequences (p > 0.05). The mean total spleen volume at each scan point for both the T_1_ and T_2_ images were compared for the infected and control groups (Fig 3). For the control group, there was no significant difference seen between the mean volume at any of the scan points (p > 0.05). For the infected group of mice, there was a significant increase in the spleen volume between day 0 and all infection time points (p < 0.005). From the T_1_ data, the mean volume of the spleen increased from 82 ± 12 mm^3^ before infection to a maximum of 1783 ± 110 mm^3^ at day 21 pi, when the infection had entered the late stage. There was no significant difference between the mean volume at day 28 pi (1483 ± 20 mm^3^) and day 14 (1339 ± 140 mm^3^) or day 21 pi. The same trend was seen in the data collected using the T_1_ weighted sequence. A full comparison of the volumes for both the control group and infected group are shown in S1–S4 Tables.

### Histology

#### Gross findings

**Fig 4:**
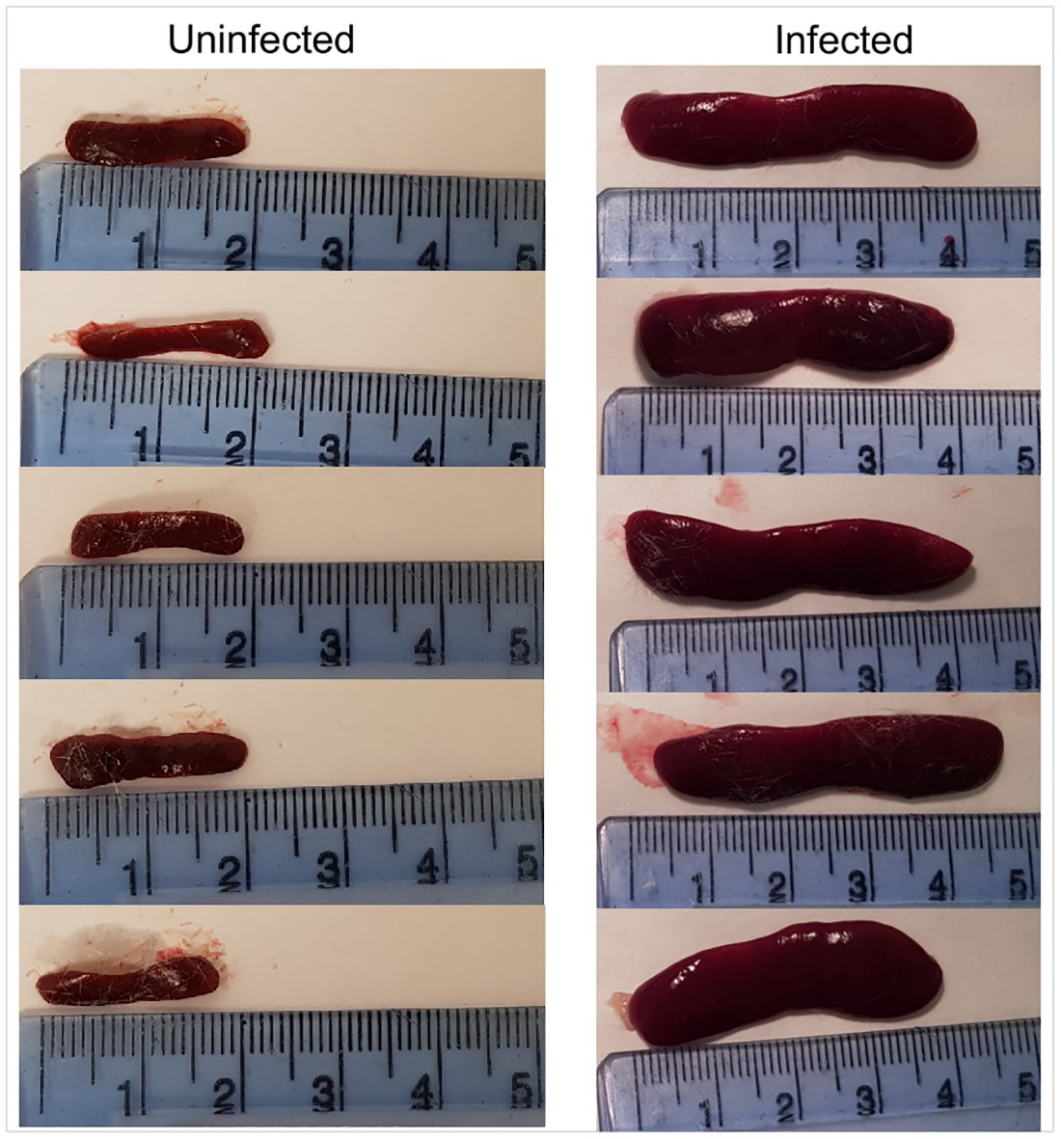
Infected and control group spleens. Spleens were taken at the end of the experiment on day 28 pi, weighed and photographed. A clear difference can be seen between the infected and control spleens, with the infected spleens appearing swollen and approximately double the length of the uninfected spleens.

At the end of the study, spleens were removed and weighed. An overt increase in spleen size was apparent in the infected animals (Fig 4). The mean spleen weight of the control group was 0.094 ± 0.007g, representing 0.27% of the body weight (35 ± 2g). The mean volume for the spleen calculated from the MR images was 0.13 ± 0.01 mm^3^. By using Eq 1, a mean mass of 0.149 ± 0.003g from the T_1_ data and 0.156 ± 0.004g from the T_2_ data was found. This was significantly higher than the gross weight (p = 0.002). For the infected group, the mean spleen weight was 1.51 ± 0.04g. This was calculated to be 4.1% of the mean body weight (37 ± 5g). The mean spleen volume of the infected mice at day 28 pi was 1.5 ± 0.3mm^3^ which gave a mass of 1.62 ± 0.06g from both the T_1_ and T_2_ datasets when using Eq 1. This value was not significantly different from the mean spleen weight (p = 0.554).

### Histopathology

**Fig 5:**
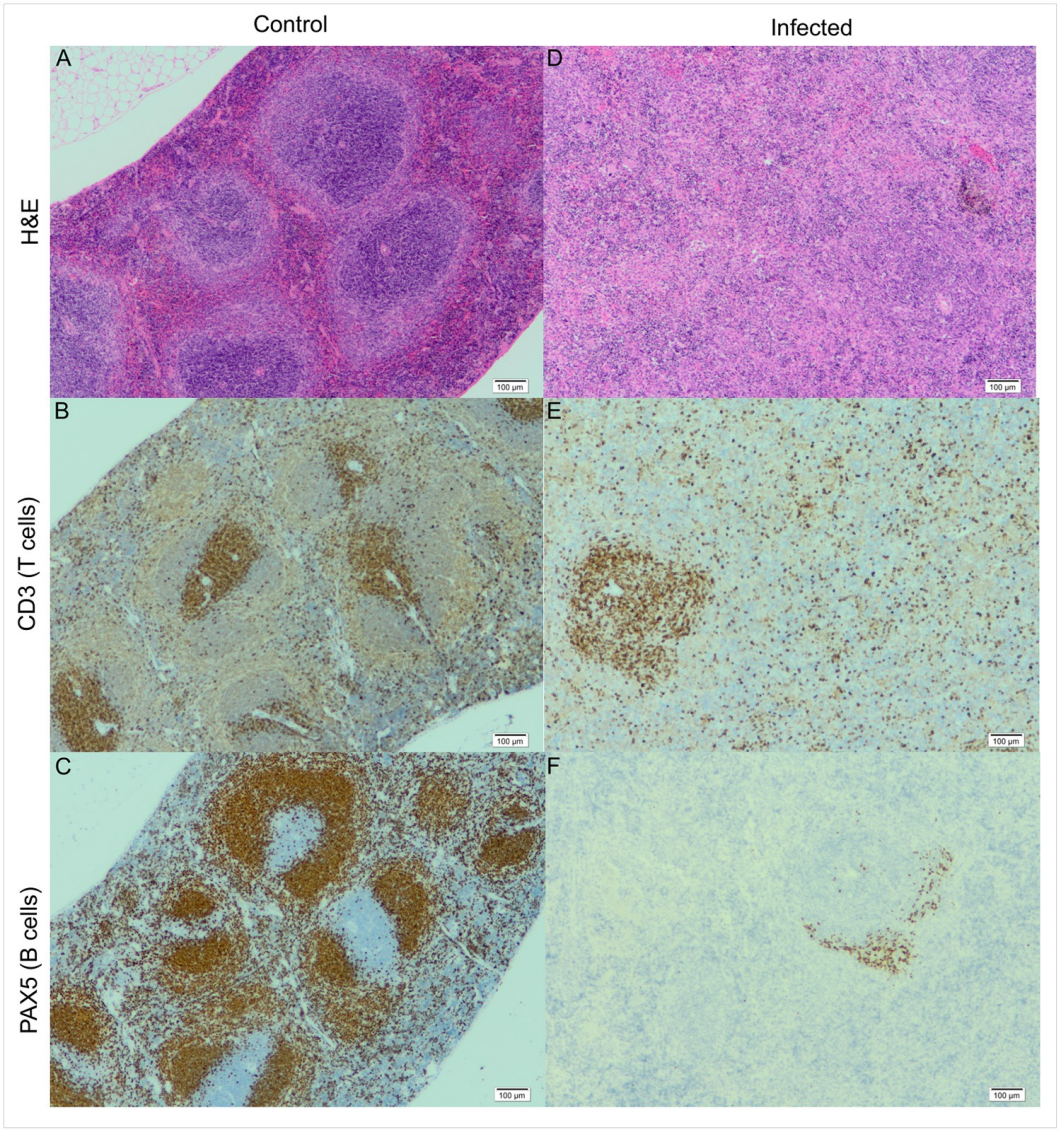
Histology of the infected and control spleen. Three stains (H&E, CD3 [T cell marker] and Pax5 [B cell marker]) were used to examine infected spleens (right) and uninfected spleens from the control group (left). A breakdown in the distinction between the red and white pulp can be seen in the infected group (D) compared to spleens from uninfected animals (A). The T cells are more diffuse in the spleen (C & D), with almost a complete loss of B cells for the infected mice (E & F). Images taken using x40 objective.

Histopathological analysis was carried out on the spleens from the infected and control groups after the final scan point (day 28 pi). H&E staining showed a separation of the red pulp and white pulp as expected in the uninfected mice (Fig 5). A clear difference was seen in the architecture of the spleen in infected mice. An obvious breakdown in the distinction between the red pulp and white pulp was apparent. The central arteries remained visible; however, the follicles were indistinct with diffuse margins. Numerous T cells were found within the periarteriolar lymphoid sheaths (PALS) surrounding the central artery, with some T cells extending into the red pulp and marginal zone in uninfected mice. Fewer T cells were seen around the central artery in the infected mice, and these appeared more diffusely spread. Staining for B cells demonstrated a large number present in the follicles and marginal zone of the control group, with B cells also detected in the surrounding red pulp. A near complete lack of B cells was observed in the infected mice with only a small number detected in the follicles and marginal zone and almost no B cells in the red pulp.

### Haematology

#### Infected group

A comparison of cell counts was made between day 0 (before infection) and day 28 pi (Table 1). There was a significant decrease (p<0.05) for the red blood cell (RBC) count, haemoglobin (Hb) and haematocrit (HCT %), with the values below the described normal range [14, 15]. There was a significant increase in the number of neutrophils and monocytes at day 28 compared with day 0 pi (p = 0.024 and p = 0.049 respectively). There was no change in the white blood cell (WBC) count at either time point, with a value of 5.4 ± 1 x10^3^ WBC / μL and 5.6 ± 1 x10^3^ WBC / μL at day 0 and day 28 pi respectively.

**Table 1:** Cell counts for the infected and control spleens. Haematological results. The cell counts from the blood samples taken from the infected and control experimental groups at day 0 (before infection) and day 28 post infection. There was a significant difference in all cell counts apart from lymphocytes and white blood cells between day 0 and day 28 in the infection group. In the control group between day 0 and 28 there was a significant difference (p < 0.05) for the white blood cells and the lymphocytes, but these values were still in the normal literature range. Significance is denoted with the blue box. Significance where the values are still in normal range are denoted by the green box and (*).

	Infected		Control		Normal Range
	Day 0	Day 28	Day 0	Day 28	
RBC (x10^6^/μL)	10 ± 0.8	5.8 ± 0.4	8.2 ± 0.7	9.1 ± 0.3	7–11
Hb (g/dl)	15.0 ± 1.5	7.8 ± 0.5	13.4 ± 1.1	13.9 ± 0.2	13.2–16.4
HCT (%)	42.2 ± 3	29.3 ± 2	38.8 ± 3	38.0 ± 1	35–52
WBC (x10^3^/μL)	5.4 ± 1	5.6 ± 1	7.8 ± 2	2.0 ± 0.4 (*)	2–10
Neutrophils (x10^3^/μL)	0.421 ± 0.09	1.65 ± 0.04 (*)	0.36 ± 0.01	0.14 ± 0.005	0.04–2
Lymphocytes (x10^3^/μL)	5 ± 1	3.2 ± 1	7.4 ± 1	1.7 ± 0.4 (*)	1.4–7
Monocytes (x10^3^/μL)	0.05 ± 0.02	0.61 ± 0.2	8.2x10^-3^ ± 7x10^-5^	0.049 ± 0.01	0–0.02

#### Control group

Comparison of blood samples taken at day 0 and day 28 pi in the control group were examined. (Table 1). There were no significant differences between the two time points for the following measurements: RBC, Hb, HCR, neutrophils, and monocytes (p > 0.05). There was a significant decrease (p < 0.05) of white blood cells and lymphocytes, but the values were within the normal literature ranges [14, 15].

#### Comparison of both groups

Comparison of the cell counts for both the infected and control groups before infection saw no significant difference between them (p > 0.05). For infected mice at day 28 pi there was, except for lymphocytes, a significant difference between all cell counts.

## Discussion

This study examined splenomegaly of trypanosome infected mice using non-invasive serial imaging. By using MRI, for the first time, changes in spleen volume in a single group of mice were charted over the course of infection. Furthermore, this study was the first to examine splenomegaly in detail for the GVR35 strain.

The volume of the spleen was measured by using two different MR techniques, T_1_ and T_2_ weighting imaging. These can provide different contrast between the tissue in the body depending on the properties of the tissue. No major differences were expected to be seen between the T_1_ and T_2_ images for the purposes of this study, but both were used to explore which provided the best contrast for measuring the volume of the spleen. Although both provided suitable data for accurately measuring the volume of spleen, with no significant difference (p > 0.05) between the values obtained from both methods for the infected and control groups, the T_2_ weighted images provided the best contrast. Using thirty slices covering the whole body of the mouse, this allowed the full volume of the spleen to be assessed.

The MRI data demonstrated that there was a significant increase in the volume of the spleen in the infected animals from day 7pi. At this time point post-infection splenomegaly cannot be detected by physical examination of the mouse. The spleen continued to increase in volume throughout both the early and late stages of the infection. In this model the late-stage of infection is reached by day 21-post infection when a cure cannot be achieved using drugs that do not cross the blood brain barrier [2]. No significant differences were detected between the spleen volume at day 14 and day 28 pi and day 21 and 28 pi. This suggests that splenomegaly development may differ in the early and late stages of the GVR35 infection, with a slight trend towards resolution occurring by day 28 pi. The mean spleen mass calculated from the MR images (1.62 ± 0.06g) was not significantly different from the gross weight of the spleens taken at the end of the experiment (1.51 ± 0.04g).

This study demonstrates that MR scanning is a viable technique for accurately measuring changes in the spleen volume, both in HAT and, potentially, other diseases that result in changes in organ size. This method also allows the tracking of splenomegaly in individual animals throughout the infection and could be a valuable research tool for various studies of HAT or splenomegaly. Furthermore, we can look at changes in the volume of the spleen and not just the weight, allowing more information about the changes in the spleen to be examined. Comparison of the spleen mass from the control group to the gross weight found that the MR mass was significantly larger than the recorded weight (p < 0.05). This may be due to the difficulty in identifying the boundary of the uninfected spleen, due to the small size of the organ in the images (the spleen is less than 0.3% of the body weight of the mouse). More advanced imaging analysis may be useful in future studies when identifying the spleen boundaries in a normal spleen. The purpose of the study however, which was to identify and track splenomegaly, means that this is not a major problem, and the use of MRI has clearly been shown to be an important method for non-invasive identification of splenomegaly.

Further examination of splenomegaly in GVR35 infected mice involved haematological and histological analysis. Haematology was undertaken to examine if changes in spleen MRI could be directly compared to changes in the blood. Unfortunately, clotting issues prevented the samples in-between day 0 and the end point being successfully processed. Comparison of blood samples taken at day 28 pi and before infection demonstrated anaemia in all subjects, with reduced RBC count, haemoglobin, and haematocrit counts. This was expected and has been seen previously in several studies. Amole et al. reported anaemia in both the acute and chronic stages of the disease [8]. In a study on rats infected with *T*.*b*. *brucei*, a significant drop was seen in the RBC count and Hb concentration, again indicating anaemia [16]. A significant increase in neutrophils and monocytes was found, with no overall change in the total white blood cell count. Deleeuw et al. found a similar significant increase of neutrophils and monocytes in *T*.*b*. *brucei* infected spleens [17]. All cell counts fell within normal values for the control animals, with any small changes attributed to natural fluctuations.

Histological examination of spleens showed clear changes between the infected and uninfected groups. The control group demonstrated normal architecture with distinction between the red and white pulp as expected for an uninfected mouse. T cells and B cells were seen in the pulp and marginal areas. The infected spleens appeared grossly enlarged and congested, with a loss of distinction between the red and white pulp apparent. Fewer, more diffusely spread, T cells were seen in the spleen following infection and B cell staining revealed an almost complete loss in these cells in the spleen. This could be due to plasma cell transformation with plasma cell hyperplasia being reported following trypanosome infection [18], and more recently for *T*. *evansi* [19] but this requires further confirmation.

Splenomegaly has been examined in other HAT models, with varying changes in spleen weight reported at the endpoint of the experiment. Examining spleen changes in other strains, Amole et al. reported splenomegaly at day 8 pi with a spleen weight of 0.85g 30 days after infection [8] using the TREU 667 strain of *Trypanosoma brucei*. They saw a smaller change in spleen size compared to this study with the uninfected controls having a spleen weight of 0.23g. This measured as a change from 0.8% of body weight to 2.4% of body weight after 30 days of infection. Our study found a change from 0.27% of body weight to 4.1% of body weight with the infected spleens being an average of 16 times larger than those of the control group. Similar results have been reported with Anosa et al. measuring a 25-fold increase in spleen weight, a rise from 0.026g to 0.673g, in deer mice infected with *T*. *brucei* EATRO 110 [9]. An increase in spleen weight of up to 30 times was noted by Murray et al. who attributed the change to a marked rise in plasma cell hyperplasia [7]. More recently, mice infected with the *T*. *brucei* strains TREU927 and STIB247 showed a weight difference from around 0.13g to 0.7g and 0.9g respectively, 10 days after infection [20], and researchers from Nigeria, Egbe-Nwiyi et al. found in rats infected with *T*.*b*. *brucei* or *T*.*b*. *congolense*, the infected spleens increased to four times the weight of the control spleens after approximately 15 and 30 days of infection respectively [21]. The differences in the weights of the spleen between the different *T*.*b*. strains demonstrates the need for a non-invasive method to be able to clearly examine changes, especially when testing treatments or studying new pathogens. It also means more unusual or unexpected changes can be more readily explored. For example, Morrison et al. measured the spleen weight increasing twenty fold when compared between day 4 and day 24 pi [22]. Morrison found that the spleen weight remained constant after day 24 pi and didn’t continue increasing, like other studies have shown. These findings could be missed when only looking at one end point, demonstrating the usefulness of serial scanning. This may reflect the lack of increases in spleen volume at day 28 pi found in the current study. This further suggests that if the infected group had continued, there may not have been any further significant changes in the weight of the spleen since no significant changes in volume were detected between day 21 and day 28 pi.

Overall, we have demonstrated that pre-clinical MRI can be useful in the research of HAT or similar diseases. The experiments performed in this study are standard MR scans that can be performed using a simple setup in any animal MR facility. Although the authors note that most biology labs will not have MRI scanners, many research institutions may have a pre-clinical imaging facility where suitable MRI scanning can take place. The aim of this research wasn’t to show that MRI could be used for diagnosis or treatment of clinical HAT cases but to be an important tool for animal researchers examining HAT. A useful application of MRI in further trypanosome studies would be in studying the effects of drug cures on the spleen and other organs. Researchers would be able to explore how a potential drug could instigate changes in the organs and track this through individual animals at multiple time points, compared to conventional research requiring the animal to be sacrificed. The techniques shown in this paper could be applied for example to the research from Dkhil et al. or Egbe-Nwiyi [23, 24] examining the changes in splenomegaly in trypanosome infected mice following treatment. Furthermore, it allows easy quantitative measurement of the spleen volume, which is not explored as readily as the change in spleen mass and the opportunity to explore changes between male and female mice additionally.

## Conclusion

We have successfully imaged splenomegaly in a group of trypanosome infected mice over the course of a 28-day infection. The results show that the average spleen volume increased by approximately 2400% at day 21 pi, with the spleen decreasing slightly in volume by day 28 pi. The study has provided new insights into splenomegaly in the GVR-35 strain and is the first study to track spleen volume in individual animals as well as demonstrating the importance of MRI as a research tool to facilitate the investigation of organ changes over time.

## Supporting information

S1 Table Comparison of mean spleen volume calculated using T_1_ data for the infected mouse group (n = 6). The data show a significant (p < 0.05) increase in spleen volume between the uninfected scan point and all other time points. No significant difference between the mean spleen volume at day 14 and day 28 pi and day 21 and day 28 pi was detected. The 95% confidence intervals are noted under each p-value in brackets. The mean spleen volume ± standard error at each time point is shown.
(DOCX)

S2 Table Comparison of mean spleen volume using T_2_ data for the infected mouse group (n = 6). The data show a significant difference (p < 0.05) increase in spleen volume between the uninfected scan point and all other time points. No significant difference between the mean spleen volume at day 14 and day 28 pi and day 21 and day 28 pi was detected. The 95% confidence intervals are noted under each p-value in brackets. The mean spleen volume ± standard error at each time point is shown.
(DOCX)

S3 Table Comparison of the mean spleen volume from the control group using T_1_ data (n = 5). There was no significant difference (p > 0.05) between any of the time points in the control group of mice. The mean spleen volume and standard error at each time point is shown. 95% confidence intervals are noted under each p-value.
(DOCX)

S4 Table Comparison of the mean spleen volume from the control group using T_2_ data (n = 5). There was no significant difference (p > 0.05) between any of the time points in the control group of mice. The mean spleen volume and standard error at each time point is shown. 95% confidence intervals are noted under each p-value.
(DOCX)

S1 Fig T_2_ weighted images of the spleen for one healthy mouse. For one healthy mouse, all slices containing the spleen are shown from a T2 weighted scan. The typical triangular cross section is seen.
(TIF)

S2 Fig T_1_ weighted images of one HAT infected mouse. For one infected mouse, all slices containing the spleen are shown from a T1 weighted scan. The splenomegaly is clear throughout all the images, with an elongated shape and displacement of other organs seen throughout.
(TIF)

S3 Fig Labelled spleen histology. Diagram depicting the basic anatomy of the spleen. The spleen is supplied with blood via the splenic artery which branches off from the central artery. The smaller branches of the spleen are sheathed by lymphoid tissue, forming the white pulp. The red and white pulp are separated by the marginal zone.
(TIFF)
