# Supplementary material for: Serial magnetic resonance imaging of splenomegaly in the Trypanosoma brucei infected mouse
Source: PLoS Negl Trop Dis. 2022 Dec 7;16(12):e0010962. doi: 10.1371/journal.pntd.0010962 (PMC9728833; doi:10.1371/journal.pntd.0010962)
Supplement: S2 Table — The data show a significant difference (p < 0.05) increase in spleen volume between the uninfected scan point and all other time points. No significant difference between the mean spleen volume at day 14 and day 28 pi and day 21 and day 28 pi was detected. The 95% confidence intervals are noted under each p-value in brackets. The mean spleen volume ± standard error at each time point is shown. (DOCX) [file pntd.0010962.s002.docx]

S2 Table

|  | Control | Day 7 | Day 14 | Day 21 | Day 28 |
| --- | --- | --- | --- | --- | --- |
| Day 7 | p = 0.002 ( 165, 871) |  |  |  |  |
| Day 14 | p < 0.001 (918,1624) | p < 0.001 (400, 1105) |  |  |  |
| Day 21 | p < 0.001 (1303, 2009) | p < 0.001 (784, 1490) | p = 0.029 (32, 738) |  |  |
| Day 28 | p < 0.001 (1046, 1752) | p < 0.001 (527, 1233) | p = 0.813 (-225, 481) | p = 0.228 (-610, 96) |  |
| Mean Volume ± SE (mm^3^) | 84 ± 10 | 603 ± 20 | 1355 ± 80 | 1740 ± 120 | 1483 ± 110 |
