# Supplementary material for: Serial magnetic resonance imaging of splenomegaly in the Trypanosoma brucei infected mouse
Source: PLoS Negl Trop Dis. 2022 Dec 7;16(12):e0010962. doi: 10.1371/journal.pntd.0010962 (PMC9728833; doi:10.1371/journal.pntd.0010962)
Supplement: S3 Table — There was no significant difference (p > 0.05) between any of the time points in the control group of mice. The mean spleen volume and standard error at each time point is shown. 95% confidence intervals are noted under each p-value. (DOCX) [file pntd.0010962.s003.docx]

S3 Table

|  | Control | Day 7 | Day 14 | Day 21 | Day 28 |
| --- | --- | --- | --- | --- | --- |
| Day 7 | p = 0.996 (-29.8, 37.7) |  |  |  |  |
| Day 14 | p = 0.961 (-41.3, 26.2) | p = 0.843 (-45.3, 22.3) |  |  |  |
| Day 21 | p = 1 (-33.7, 33.9) | p = 0.997 (-37.7, 29.9) | p = 0.96 (-26.2, 41.4) |  |  |
| Day 28 | p = 0.997 (-30.1, 37.5) | p = 1 (-34.0, 33.5) | p = 0.853 (-22.5, 45.1) | p = 0.997 (-30.1, 37.4) |  |
| Mean Volume ± SE (mm^3^) | 133 ± 6 | 137 ± 7 | 126 ± 8 | 133 ± 10 | 137 ± 6 |
