# Supplementary material for: Serial magnetic resonance imaging of splenomegaly in the Trypanosoma brucei infected mouse
Source: PLoS Negl Trop Dis. 2022 Dec 7;16(12):e0010962. doi: 10.1371/journal.pntd.0010962 (PMC9728833; doi:10.1371/journal.pntd.0010962)
Supplement: S4 Table — There was no significant difference (p > 0.05) between any of the time points in the control group of mice. The mean spleen volume and standard error at each time point is shown. 95% confidence intervals are noted under each p-value. (DOCX) [file pntd.0010962.s004.docx]

S4 Table

|  | Control | Day 7 | Day 14 | Day 21 | Day 28 |
| --- | --- | --- | --- | --- | --- |
| Day 7 | p = 0.997 (-46.7, 37.1) |  |  |  |  |
| Day 14 | p = 0.880 (-55.0, 28.8) | p = 0.975 (-50.1, 33.6) |  |  |  |
| Day 21 | p = 0.998 (-46.3, 37.5) | p = 1.000 (-41.5, 42.3) | p = 0.970 (-33.2, 50.6) |  |  |
| Day 28 | p = 0.859 (-28.1, 55.7) | p = 0.677 (-23.3, 60.5) | p = 0.339 (-15.0, 68.8) | p = 0.694 (-23.7, 60.1) |  |
| Mean Volume ± SE (mm^3^) | 133 ± 6 | 137 ± 7 | 126 ± 8 | 133 ± 10 | 137 ± 6 |
