# Supplementary figures and images for: Serial magnetic resonance imaging of splenomegaly in the Trypanosoma brucei infected mouse
Source: PLoS Negl Trop Dis. 2022 Dec 7;16(12):e0010962. doi: 10.1371/journal.pntd.0010962 (PMC9728833; doi:10.1371/journal.pntd.0010962)

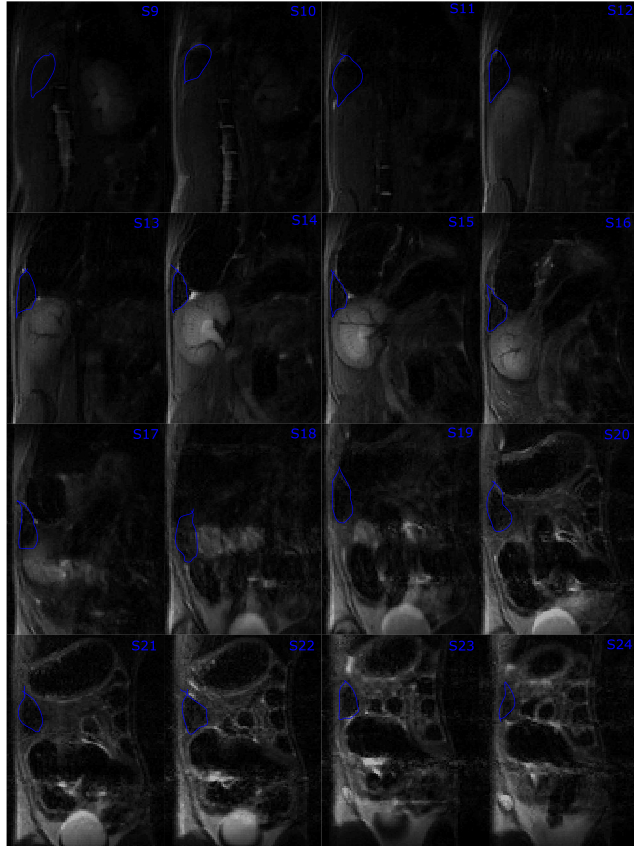

Supplement: S1 Fig — For one healthy mouse, all slices containing the spleen are shown from a T2 weighted scan. The typical triangular cross section is seen. (TIF) [file pntd.0010962.s005.tif]

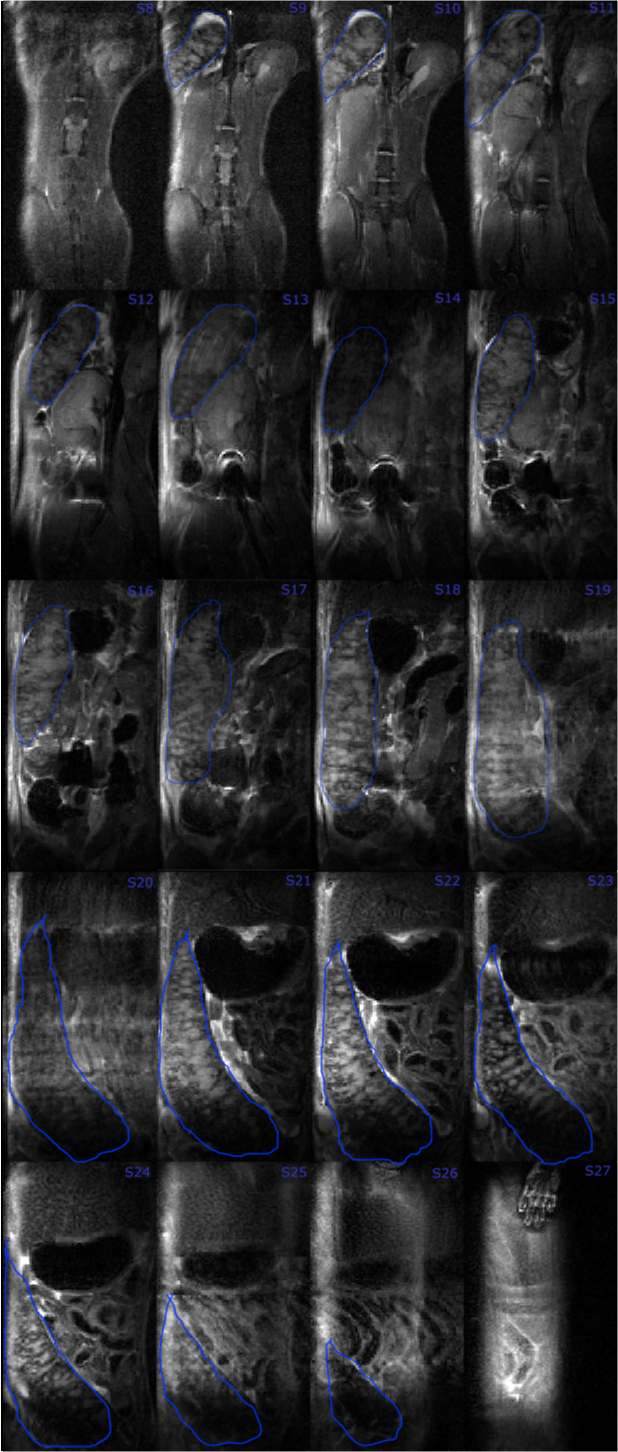

Supplement: S2 Fig — For one infected mouse, all slices containing the spleen are shown from a T1 weighted scan. The splenomegaly is clear throughout all the images, with an elongated shape and displacement of other organs seen throughout. (TIF) [file pntd.0010962.s006.tif]

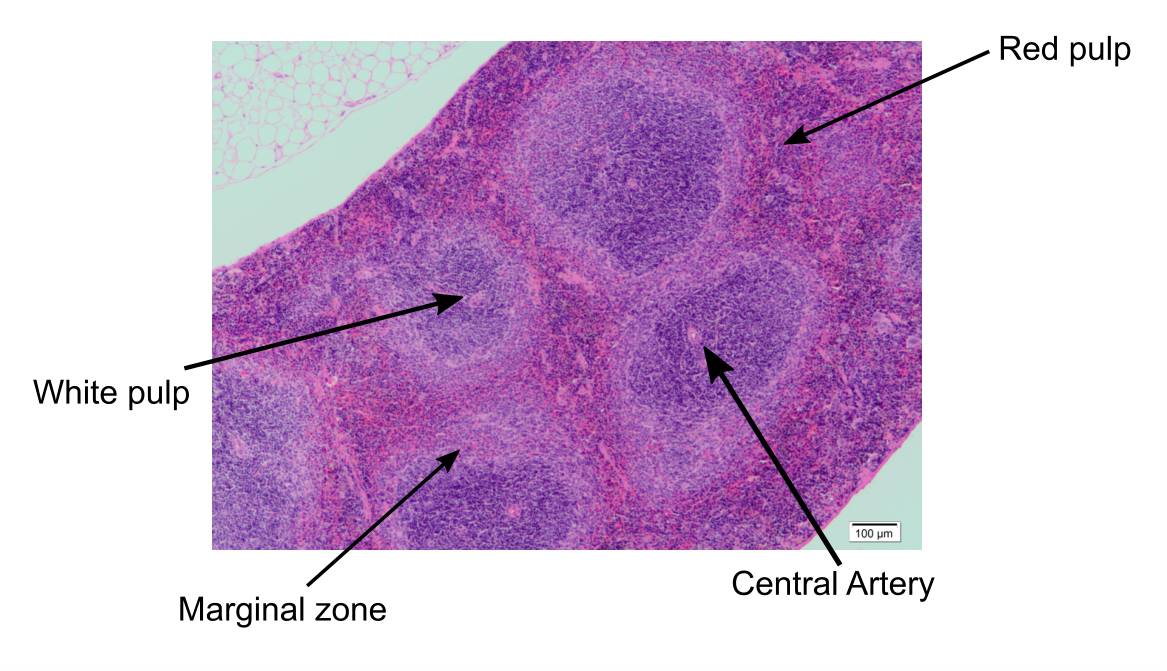

Supplement: S3 Fig — Diagram depicting the basic anatomy of the spleen. The spleen is supplied with blood via the splenic artery which branches off from the central artery. The smaller branches of the spleen are sheathed by lymphoid tissue, forming the white pulp. The red and white pulp are separated by the marginal zone. (TIFF) [file pntd.0010962.s007.tiff]
